# Brain-Derived Neurotrophic Factor Ameliorates Brain Stem Cardiovascular Dysregulation during Experimental Temporal Lobe Status Epilepticus

**DOI:** 10.1371/journal.pone.0033527

**Published:** 2012-03-19

**Authors:** Ching-Yi Tsai, Julie Y. H. Chan, Kuei-sen Hsu, Alice Y. W. Chang, Samuel H. H. Chan

**Affiliations:** 1 Center for Translational Research in Biomedical Sciences, Kaohsiung Chang Gung Memorial Hospital, Kaohsiung, Taiwan, Republic of China; 2 Department of Pharmacology, National Cheng Kung University, Tainan, Taiwan, Republic of China; Université de Montréal, Canada

## Abstract

**Background:**

Status epilepticus (SE) is an acute, prolonged epileptic crisis with a mortality rate of 20–30%; the underlying mechanism is not completely understood. We assessed the hypothesis that brain stem cardiovascular dysregulation occurs during SE because of oxidative stress in rostral ventrolateral medulla (RVLM), a key nucleus of the baroreflex loop; to be ameliorated by brain-derived neurotrophic factor (BDNF) via an antioxidant action.

**Methodology/Principal Findings:**

In a clinically relevant experimental model of temporal lobe SE (TLSE) using Sprague-Dawley rats, sustained hippocampal seizure activity was accompanied by progressive hypotension that was preceded by a reduction in baroreflex-mediated sympathetic vasomotor tone; heart rate and baroreflex-mediated cardiac responses remained unaltered. Biochemical experiments further showed concurrent augmentation of superoxide anion, phosphorylated p47^phox^ subunit of NADPH oxidase and mRNA or protein levels of BDNF, tropomyosin receptor kinase B (TrkB), angiotensin AT1 receptor subtype (AT1R), nitric oxide synthase II (NOS II) or peroxynitrite in RVLM. Whereas pretreatment by microinjection bilaterally into RVLM of a superoxide dismutase mimetic (tempol), a specific antagonist of NADPH oxidase (apocynin) or an AT1R antagonist (losartan) blunted significantly the augmented superoxide anion or phosphorylated p47^phox^ subunit in RVLM, hypotension and the reduced baroreflex-mediated sympathetic vasomotor tone during experimental TLSE, pretreatment with a recombinant human TrkB-Fc fusion protein or an antisense *bdnf* oligonucleotide significantly potentiated all those events, alongside peroxynitrite. However, none of the pretreatments affected the insignificant changes in heart rate and baroreflex-mediated cardiac responses.

**Conclusions/Significance:**

We conclude that formation of peroxynitrite by a reaction between superoxide anion generated by NADPH oxidase in RVLM on activation by AT1R and NOS II-derived NO leads to a reduction in baroreflex-mediated sympathetic vasomotor tone during experimental TLSE; to be ameliorated by the upregulated BDNF/TrkB signaling via inhibition of p47^phox^ phosphorylation. This information offers a new vista in devising therapeutic strategy towards minimizing mortality associated with TLSE.

## Introduction

Status epilepticus (SE) is an acute, prolonged epileptic crisis and is a common, life-threatening neurological disorder [1], [2]. As a medical emergency, SE has a mortality rate that ranges between 20 and 30% [3], [4]. A majority of the studies that addresses the mechanisms that underlie the mortality associated with SE has included the heart as the primary target. Thus, lethal cardiac arrhythmias [4], [5] or cardiac damage that increases the susceptibility to arrhythmia [6], [7] after SE have been reported. Two pieces of information further implicate a potential role for brain stem cardiovascular regulation in SE-linked mortality. First, as SE continues, the prominent elevation in systemic arterial pressure exhibited at the beginning of SE shifts to a decrease to levels below baseline [8]. Second, seizures activate areas in the medulla oblongata [9], [10], including nucleus tractus solitarii (NTS) and rostral ventrolateral medulla (RVLM), that are associated with brain stem cardiovascular regulation [11], [12].

The most fundamental mechanism in brain stem cardiovascular regulation exists in the form of baroreflex, which provides a rapid negative feedback mechanism that dampens fluctuations in circulatory parameters induced by environmental insults [11], [12]. The carotid sinus and aortic depressor nerves convey primary baroreceptor afferent information to NTS. Outputs from NTS modulate the activity of parasympathetic premotor neurons in the dorsal motor nucleus of vagus or nucleus ambiguus that mediate reflex control of the heart; as well as the reticulospinal vasomotor neurons in RVLM that mediate reflex adjustment of sympathetic outflow to the blood vessels. Operated under physiological conditions, baroreflex is responsible for the maintenance of stable arterial pressure (AP). Severe and even fatal consequences, however, will take place under pathological conditions when the baroreflex is dysregulated. Whether dysfunction of baroreflex-mediated cardiac and vasomotor responses is associated with SE, and detailed underlying cellular and molecular mechanisms, are wanting.

The reactive oxygen species (ROS), including free radicals such as superoxide anion, are produced virtually by all aerobic cells. The production and removal of ROS are tightly controlled under physiological conditions. Nonetheless, under pathological situations, excessive production of ROS may surpass the endogenous antioxidant defense mechanisms for the degradation of ROS, leading to conditions that are referred to as oxidative stress. An increasing body of evidence suggests that oxidative stress is involved in the pathogenesis of many cardiovascular diseases [13]–[15]. In terms of brain stem cardiovascular regulation, clinical and animal studies [16], [17] demonstrated that oxidative stress impairs baroreflex sensitivity (BRS).

Superimposed on its classical trophic functions in the peripheral and central nervous system during development [18] or in synaptic activity and plasticity of mature neurons [19], brain-derived neurotrophic factor (BDNF) is now known to possess nontrophic actions [20]. In addition to neuroprotective effects, which defend neurons against injury and diseases [21]–[23], there are indications that BDNF possesses an antioxidant action [24], [25]. Moreover, BDNF and its receptor, tropomyosin receptor kinase B (TrkB) are distributed in brain stem nuclei that subserve cardiovascular regulation [26]–[28]. With particular relevance to the present study, our laboratory showed [29] recently that BDNF plays an active role in neural regulation of AP by maintaining ROS, particularly superoxide anion homeostasis in RVLM.

Our laboratory has developed an experimental model of SE that is coupled with temporal lobe epilepsy [30]–[32], the most common form of epilepsy [33]. Based on this experimental TLSE model and employing RVLM as the target neural substrate, the present study assessed the hypothesis that oxidative stress in RVLM, leading to brain stem cardiovascular dysregulation occurs during TLSE; to be ameliorated by BDNF via an antioxidant action. Our complementary physiological, pharmacological and biochemical results validated this hypothesis.

## Results

### Experimental temporal lobe status epilepsy

**Figure 1:**
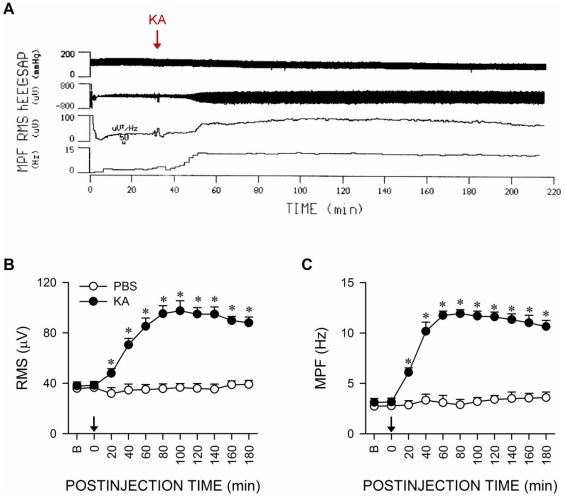
Manifestation of seizure-like hippocampal electroencephalographic activity during experimental temporal lobe status epilepticus (TLSE). Representative original tracings (A) and summary of temporal changes in root mean square (RMS) (B) or mean power frequency (MPF) (C) values of seizure-like hippocampal electroencephalographic activity recorded from the CA3 subfield on the right side on microinjection of kainic acid (KA; 0.5 nmol) or 0.1 M phosphate buffered saline (PBS, pH 7.4) unilaterally to the left CA3 area (at arrow). Values are mean ± SEM from 5–7 animals per experimental group. *P<0.05 versus PBS group at corresponding time-points in the post hoc Scheffé multiple-range test. B = preinjection baseline.

An experimental model [30]–[32] that mimics the clinical manifestations of TLSE was used. As reported previously, microinjection unilaterally of kainic acid (KA; 0.5 nmol) into the left hippocampal CA3 subfield elicited a significant and sustained buildup of seizure-like hippocampal EEG activity (Figure 1A) that can be quantified by the progressive and concomitant increase in both root mean square (Figure 1B) and mean power frequency (Figure 1C) values of hippocampal EEG signals recorded from the CA3 subfield on the right side. On the other hand, unilateral application of the solvent, phosphate-buffered saline (PBS) to the left hippocampal CA3 subfield was ineffective (Figure 1B,C). For the purpose of the present study, we routinely recorded those seizure activities for 180 min after induction.

### Reduced baroreflex-mediated sympathetic vasomotor tone during experimental TLSE

**Figure 2:**
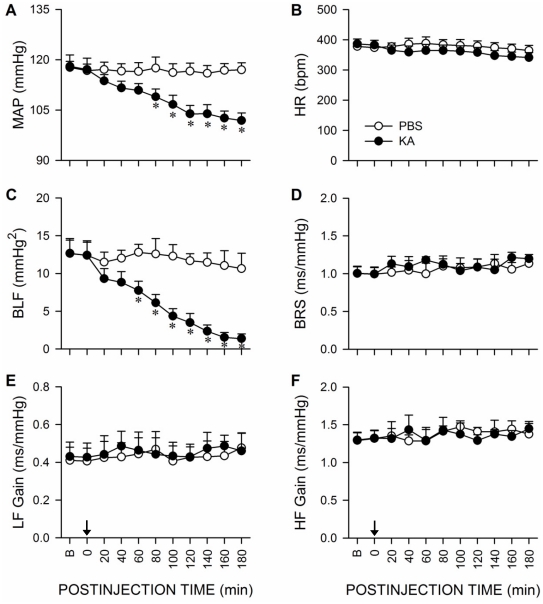
Reduced baroreflex-mediated sympathetic vasomotor tone during experimental TLSE. Temporal changes in mean arterial pressure (MAP) (A), heart rate (HR;) (B), power density of the low-frequency (BLF) component of systolic blood pressure (SBP) spectrum (C), baroreflex sensitivity (BRS) (D), and gain of transfer function between pulse interval and SBP spectra at the low-frequency (LF) (E) or high-frequency (HF) (F) bands on microinjection of KA (0.5 nmol) or PBS into the left hippocampal CA3 subfield (at arrow). Values are mean ± SEM from 5–7 animals per experimental group. *P<0.05 versus PBS group at corresponding time-points in the post hoc Scheffé multiple-range test. B = preinjection baseline.

Simultaneous evaluation of hemodynamic parameters (Figure 2) revealed that whereas mean arterial pressure (MAP) underwent a progressive reduction that became statistically significant 80 min after the induction of experimental TLSE, heart rate (HR) remained stable during the 180-min observation period. Mechanistic delineations using auto-spectral analysis of systolic blood pressure (SBP) signals indicated a progressive reduction in baroreflex-mediated sympathetic vasomotor tone [34], as denoted by a significant decrease in the power density of the low-frequency (BLF) component in the SBP spectrum, 20 min before significant hypotension took place. On the other hand, evaluation of baroreflex-mediated cardiac responses using the sequence method [35] showed that BRS exhibited insignificant alterations over 180-min after the induction of experimental TLSE. Likewise, cross-spectral analysis based on changes in the gain of transfer function between pulse interval (PI) and SBP spectra at the low-frequency (LF) and high-frequency (HF) bands, which respectively represents the influence of baroreflex-mediated sympathetic and vagal regulation on the heart [35], also showed insignificant changes. Again, unilateral microinjection of PBS into the left hippocampal CA3 subfield exerted no significant changes in all parameters examined (Figure 2).

### Oxidative stress in RVLM during experimental TLSE

**Figure 3:**
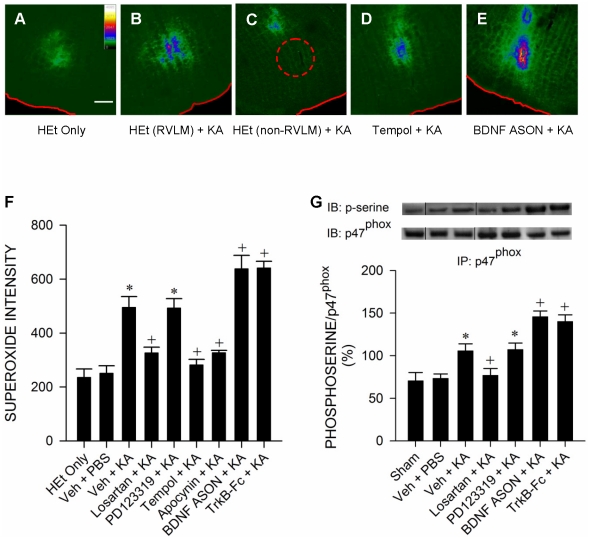
Oxidative stress in RVLM during experimental TLSE. (A–E) Representative photomicrographs showing the relative intensity (inset) of fluorescence emitted by 2-hydroxyethidium, the specific reaction product between superoxide and hydroethidine (HEt), which was microinjected into RVLM (B) or immediately outside RVLM (C) before application of KA (0.5 nmol) into the left hippocampal CA3 subfield of animals that received pretreatment by microinjection into the bilateral RVLM of distilled water or 0.1 M PBS (Veh) (B,C), tempol (D) or an antisense oligonucleotide (ASON) against *bdnf* gene (E). Note minimal fluorescence when HEt was applied to RVLM in animals that received no further experimental manipulations (A). Scale bar, 200 µm. Note that the relative position of RVLM in (C) is denoted by a circle in dashed line. (F–G) Summary of relative intensity of superoxide anion based on 2-hydroxyethidium fluorescence at RVLM (F) or the percentage of phosphoserine (p-serine) relative to p47^phox^ obtained from the cytosolic fraction of proteins extracted from RVLM that were immunoprecipitated by an anti-p47^phox^ antiserum, followed by Western blot analysis of p-serine or p47^phox^, 180 min after microinjection of KA (0.5 nmol) or PBS into the left hippocampal CA3 subfield of animals that received pretreatment by application into the bilateral RVLM of distilled water, 1% DMSO or 0.1 M PBS (Veh), losartan (2 nmol), PD123319 (2 nmol), tempol (200 pmol), apocynin (2 nmol), BDNF ASON (100 pmol) or a recombinant human TrkB-Fc fusion protein (TrkB-Fc; 1.5 pmol). Values are mean ± SEM from 5–7 animals per experimental group.*P<0.05 versus HEt alone or Veh+PBS group, and ^+^ *P*<0.05 versus Veh+KA group in the post hoc Scheffé multiple-range test. Note that in (G), dividing lines are placed on the gel images to denote groupings of images from different parts of the same gel or from different gels. Note also that since similar results were obtained from 0.1 M PBS, 1% DMSO or distilled water pretreatments, they were represented collectively by Veh in this and Figures 4–5 and 7–12 for clarity.

As the origin of the BLF component in the SBP spectrum [36], RVLM presents itself as a logical neural substrate for further biochemical evaluations. Based on the relative intensity of fluorescence emitted by hydroxyethidium [37], the specific reaction product between hydroethidine and superoxide anion, we found a significant elevation of superoxide anion level in RVLM during experimental TLSE (Figure 3B,F). The specificity of this demonstrated oxidative stress was confirmed by two observations. First, minimal amount of fluorescence was detected in animals that received microinjection of hydroethidine bilaterally into RVLM without the induction of experimental TLSE (Figure 3A,F) or in vehicle-controls (Figure 3F). Second, hydroethidine microinjected to sites immediately adjacent to RVLM in animals that were subject to experimental TLSE also exhibited minimal amount of fluorescence (Figure 3C). Pretreatment with microinjection bilaterally of a superoxide dismutase mimetic [38], tempol (200 pmol) into RVLM significantly blunted the elevation of superoxide anion level in RVLM (Figure 3D,F). We further showed that one of the sources of superoxide anion is NADPH oxidase. Microinjection bilaterally of a specific antagonist of NADPH oxidase [39], apocynin (2 nmol) into bilateral RVLM antagonized significantly the augmented level of superoxide anion (Figure 3F). In addition, co-immunoprecipitation experiments demonstrated a significant increase in phosphorylated p47^phox^ subunit of NADPH oxidase in RVLM during experimental TLSE (Figure 3G) when compared to sham-controls or vehicle-controls.

### Oxidative stress in RVLM underlies the reduced baroreflex-mediated sympathetic vasomotor tone during experimental TLSE

**Figure 4:**
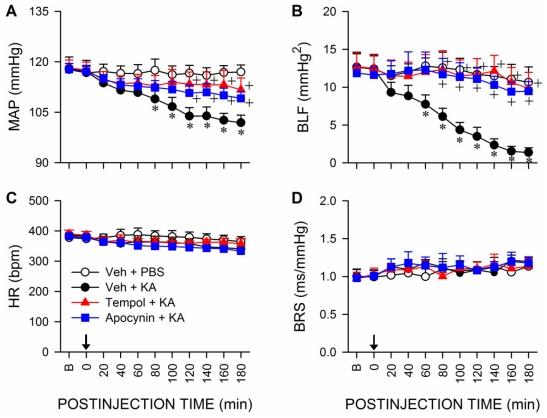
Oxidative stress in RVLM underlies the reduced baroreflex-mediated sympathetic vasomotor tone during experimental TLSE. Temporal changes in MAP (A), power density of the BLF component (B), HR (C) or BRS (D) after microinjection of KA (0.5 nmol) or PBS into the left hippocampal CA3 subfield (at arrow) of animals that received pretreatment by application into the bilateral RVLM of distilled water or 1% DMSO (Veh), tempol (200 pmol) or apocynin (2 nmol). Values are mean ± SEM from 5–7 animals per experimental group.*P<0.05 versus Veh+PBS group, and ^+^ *P*<0.05 versus Veh+KA group at corresponding time-points in the post hoc Scheffé multiple-range test. B = preinjection baseline.

**Figure 5:**
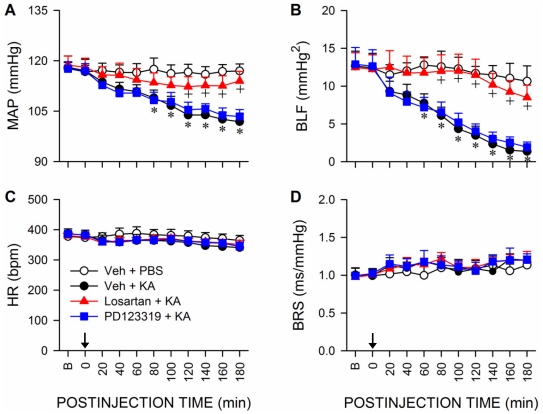
Activation of angiotensin AT1 receptor subtype in RVLM underlies the reduced baroreflex-mediated sympathetic vasomotor tone during experimental TLSE. Temporal changes in MAP (A), power density of the BLF component (B), HR (C) or BRS (D) after microinjection of KA (0.5 nmol) or PBS into the left hippocampal CA3 subfield (at arrow) of animals that received pretreatment by application into the bilateral RVLM of distilled water (Veh), losartan (2 nmol) or PD123319 (2 nmol). Values are mean ± SEM from 5–7 animals per experimental group.*P<0.05 versus Veh+PBS group, and ^+^ *P*<0.05 versus Veh+KA group at corresponding time-points in the post hoc Scheffé multiple-range test. B = preinjection baseline.

Pretreatment by microinjection of tempol (200 pmol) or apocynin (2 nmol), but not their solvents, into bilateral RVLM significantly blunted the reduction in MAP and power density of BLF component during experimental TLSE (Figure 4). Both pretreatments, however, exerted minimal effects on the insignificant changes in HR or BRS (Figure 4). We further investigated the role of angiotensin AT1 receptor subtype (AT1R) at RVLM in the reduced baroreflex-mediated sympathetic vasomotor tone because previous work from our laboratory [29], [40] showed that activation of AT1R results in oxidative stress in RVLM. In addition to significantly blunted the elevated superoxide anion levels (Figure 3F) or phosphorylated p47^phox^ subunit (Figure 3G) in RVLM, microinjection of an AT1R antagonist [41], losartan (2 nmol) bilaterally into RVLM resulted in an antagonism of the depressed MAP and BLF power that was reminiscent of tempol or apocynin pretreatment (Figure 5), without affecting HR or BRS. On the other hand, pretreatment with an equimolar dose of an AT2R antagonist [42], PD123319 (2 nmol), similar to vehicle-control, was ineffective against the elicited oxidative stress (Figure 3F,G) or cardiovascular events (Figure 5).

### Upregulation of BDNF and TrkB exerts an antioxidant action in RVLM during experimental TLSE

**Figure 6:**
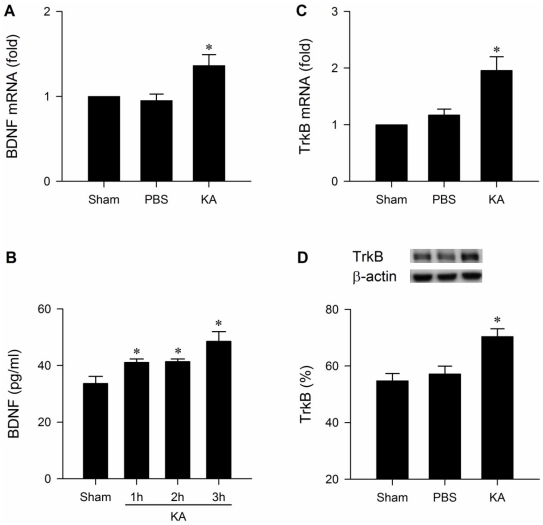
Upregulation of BDNF and TrkB in RVLM during experimental TLSE. Fold-changes in BDNF or TrkB mRNA against sham-control detected by real-time PCR (A,C), temporal changes in tissue levels of BDNF detected by ELISA (B) or TrkB detected by Western blot analysis (D) in tissues collected from bilateral RVLM 180 min (A,C,D) or 1, 2 or 3 h (B) after microinjection of KA (0.5 nmol) or PBS into the left hippocampal CA3 subfield. Values are mean ± SEM of triplicate analyses from 5–6 animals per experimental group. *P<0.05 versus sham-control or PBS group in the post hoc Scheffé multiple-range test.

Quantification by ELISA revealed a progressive elevation of BDNF level in RVLM during experimental TLSE (Figure 6B). Real-time PCR further showed that this elevation may result from transcriptional upregulation of *bdnf* gene (Figure 6A). There was also an augmentation of serum level of BDNF (sham: 32.6±2.3 pg/ml; TLSE: 48.6±1.9 pg/ml; mean ± SEM, P<0.05, n = 5–7 animals per group). Likewise, TrkB underwent an augmentation at the mRNA (Figure 6C) or protein (Figure 6D) level in RVLM. More importantly, pretreatment with microinjection into bilateral RVLM of a recombinant human TrkB-Fc fusion protein (TrkB-Fc; 1.5 pmol) [29], which sequesters endogenously released TrkB ligands and blocks TrkB [43], but not the solvent, significantly potentiated the already elevated superoxide anion levels (Figure 3F) or phosphorylated p47^phox^ subunit (Figure 3G) in RVLM after the induction of experimental TLSE. Knock-down of *bdnf* gene with an antisense oligonucleotide (100 pmol) similarly potentiated the augmented superoxide anion level (Figure 3E,F) and activation of NADPH oxidase in RVLM (Figure 3G).

### Upregulation of BDNF and TrkB in RVLM amelioriates the reduced baroreflex-mediated sympathetic vasomotor tone during experimental TLSE

**Figure 7:**
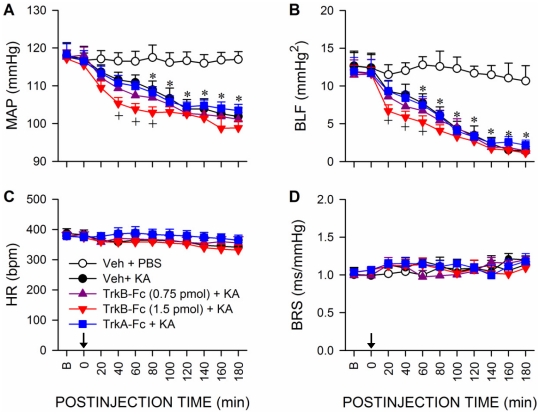
Upregulation of TrkB in RVLM ameliorates the reduced baroreflex-mediated sympathetic vasomotor tone during experimental TLSE. Temporal changes in MAP (A), power density of the BLF component (B), HR (C) or BRS (D) after microinjection of KA (0.5 nmol) or PBS into the left hippocampal CA3 subfield (at arrow) of animals that received pretreatment by application into the bilateral RVLM of 0.1 M PBS (Veh), TrkB-Fc (0.75 or 1.5 pmol) or TrkA-Fc (1.5 pmol). Values are mean ± SEM from 5–7 animals per experimental group.*P<0.05 versus Veh+PBS group, and ^+^ *P*<0.05 versus Veh+KA group at corresponding time-points in the post hoc Scheffé multiple-range test. B = preinjection baseline.

**Figure 8 pone-0033527-g008:**
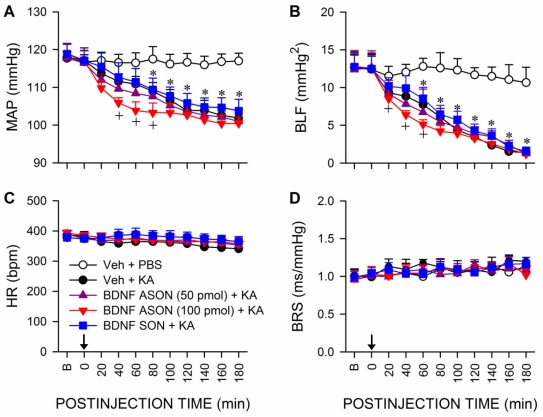
Upregulation of BDNF in RVLM ameliorates the reduced baroreflex-mediated sympathetic vasomotor tone during experimental TLSE. Temporal changes in MAP (A), power density of the BLF component (B), HR (C) or BRS (D) after microinjection of KA (0.5 nmol) or PBS into the left hippocampal CA3 subfield (at arrow) of animals that received pretreatment by application into the bilateral RVLM of distilled water (Veh), an antisense (ASON; 50 or 100 pmol) or sense oligonucleotide (SON) against *bdnf* gene (100 pmol). Values are mean ± SEM from 5–7 animals per experimental group.*P<0.05 versus Veh+PBS group, and ^+^ *P*<0.05 versus Veh+KA group at corresponding time-points in the post hoc Scheffé multiple-range test. B = preinjection baseline.

Pretreatment by applying TrkB-Fc (1.5 pmol) (Figure 7) or an antisense oligonucleotide against *bdnf* gene (100 pmol) (Figure 8) to RVLM also significantly exacerbated the reduction in MAP and power density of BLF component of SBP signals during experimental TLSE. On the other hand, a lower dose of TrkB-Fc (0.75 pmol; Figure 7) or antisense oligonucleotide against *bdnf* gene (50 pmol; Figure 8) was ineffective, as were control pretreatments (Figures 7 and 8) with a recombinant human TrkA-Fc fusion protein (TrkA-Fc; 1.5 pmol), a sense oligonucleotide against *bdnf* gene (100 pmol) or the solvents. All pretreatments also exerted minimal effects on the insignificant changes in HR or BRS (Figures 7 and 8).

### BDNF and TrkB reduce the upregulated peroxynitrite in RVLM during experimental TLSE

**Figure 9 pone-0033527-g009:**
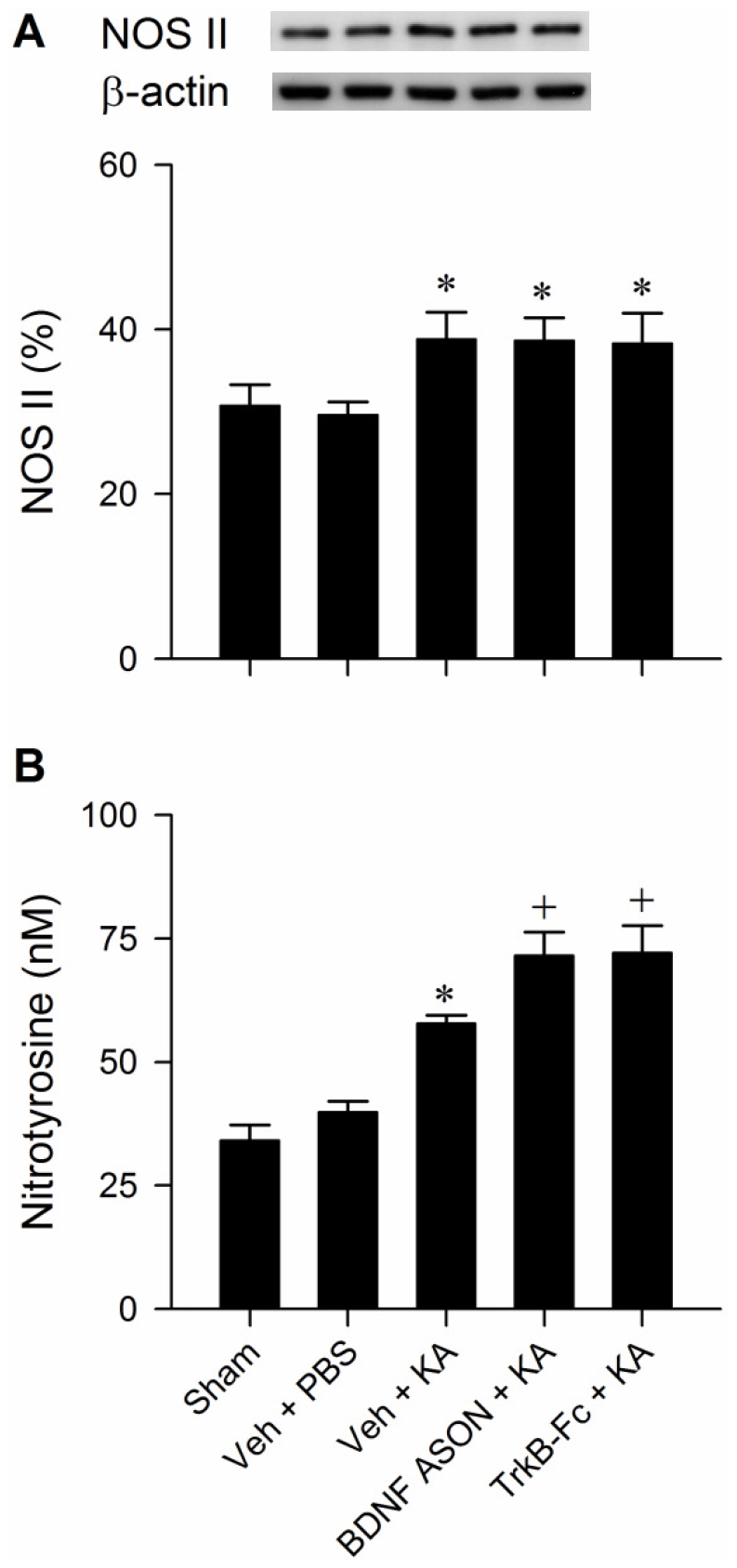
BDNF and TrkB reduce the upregulated peroxynitrite levels in RVLM during experimental TLSE. Illustrative gels or summary of changes in NOS II detected by Western blot analysis (A) or nitrotyrosine (experimental index for peroxynitrite) detected by ELISA (B) in samples collected from bilateral RVLM of sham-control or 180 min after microinjection of KA (0.5 nmol) or PBS into the left hippocampal CA3 subfield of animals that received pretreatment by application into the bilateral RVLM of distilled water or 0.1 M PBS (Veh), BDNF ASON (100 pmol) or TrkB-Fc (1.5 pmol). Values are mean ± SEM of triplicate analyses on individual samples obtained from 5–6 animals per experimental group. *P<0.05 versus sham-control or Veh+PBS group, and ^+^ *P*<0.05 versus Veh+KA group in the post hoc Scheffé multiple-range test.

Results from Western blot analysis showed a significant increase in nitric oxide synthase II (NOS II) expression (Figure 9A); and ELISA revealed a significant elevation in nitrotyrosine, an experimental index for peroxynitrite (Figure 9B) during experimental TLSE. Of note was that pretreatments with an antisense oligonucleotide against *bdnf* gene (100 pmol) or TrkB-Fc (1.5 pmol) further augmented nitrotyrosine expression in RVLM (Figure 9B), without affecting the elevated NOS II level (Figure 9A).

### Upregulation of BDNF and TrkB is not a consequence of oxidative stress in RVLM

**Figure 10 pone-0033527-g010:**
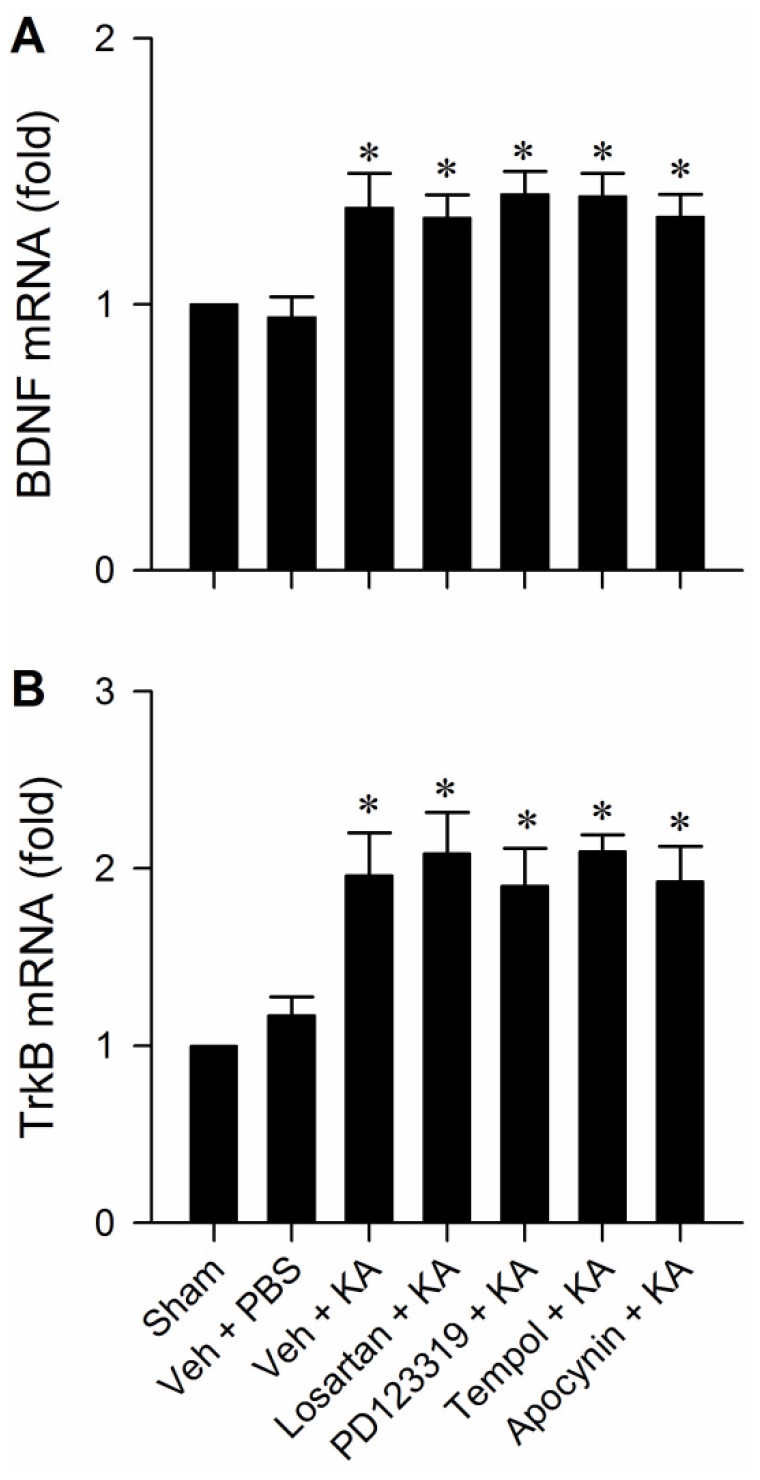
Upregulation of BDNF and TrkB is not a consequence of oxidative stress in RVLM. Fold-changes in BDNF (A) or TrkB (B) mRNA against sham-control detected by real-time PCR in tissues collected from bilateral RVLM 180 min after microinjection of KA (0.5 nmol) or PBS into the left hippocampal CA3 subfield of animals that received pretreatment by application into the bilateral RVLM of distilled water or 1% DMSO (Veh), losartan (2 nmol), PD123319 (2 nmol), tempol (200 pmol) or apocynin (2 nmol). Values are mean ± SEM of triplicate analyses from 5–6 animals per experimental group. *P<0.05 versus sham-control or PBS group in the post hoc Scheffé multiple-range test.

We reported recently [29] that chronic infusion of angiotensin II (Ang II) induces AT1R- and superoxide-dependent upregulation of BDNF in RVLM. It would therefore be of interest to delineate whether the same cellular events occur during experimental TLSE. Pretreatment with microinjection bilaterally of losartan (2 nmol), PD123319 (2 nmol), tempol (200 pmol) or apocynin (2 nmol) into RVLM, similar to vehicle-controls, did not elicit significant effects on the upregulated BDNF (Figure 10A) or TrkB (Figure 10B) mRNA in RVLM during experimental TLSE.

### BDNF and TrkB do not interact with AT1R in RVLM

**Figure 11 pone-0033527-g011:**
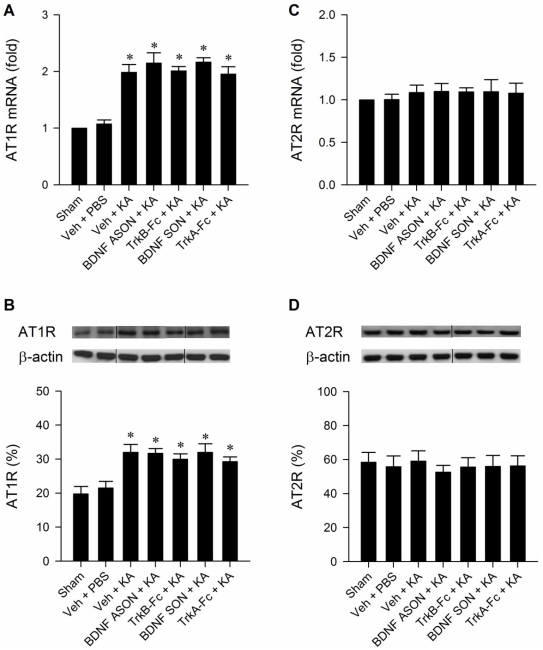
BDNF and TrkB do not interact with AT1R in RVLM. Illustrative gels or summary of fold changes in angiotensin receptor subtype 1 (AT1R) or subtype 2 (AT2R) mRNA (A,C) against sham-control detected by real-time PCR (A,C) or AT1R or AT2R protein detected by Western blot analysis (B,D) in samples collected from bilateral RVLM 180 min after microinjection of KA (0.5 nmol) or PBS into the left hippocampal CA3 subfield of animals that received pretreatment by application into the bilateral RVLM of distilled water or 0.1 M PBS (Veh), BDNF ASON or SON (100 pmol), TrkB-Fc (1.5 pmol) or TrkA-Fc (1.5 pmol). Values are mean ± SEM of triplicate analyses on individual samples obtained from 5–6 animals per experimental group. *P<0.05 versus sham-control or PBS group in the post hoc Scheffé multiple-range test. Note that in (B,D), dividing lines are placed on the gel images to denote groupings of images from different parts of the same gel or from different gels.

Real-time PCR and Western blot analysis revealed that both mRNA and protein levels of AT1R in RVLM were significantly augmented after the induction of experimental TLSE (Figure 11A,B). This induced upregulation of AT1R, however, was not affected by pretreatments with microinjection of antisense or sense oligonucleotide against *bdnf* gene (100 pmol), TrkA-Fc (1.5 pmol) or TrkB-Fc (1.5 pmol) into the bilateral RVLM (Figure 11A,B). At the same time, the mRNA and protein levels of AT2R in RVLM exhibited no significant alterations during experimental TLSE, and were similarly unaffected by the above pretreatments (Figure 11C,D).

### Glutamatergic neurotransmission in RVLM is not involved in the reduced baroreflex-mediated sympathetic vasomotor tone during experimental TLSE

**Figure 12:**
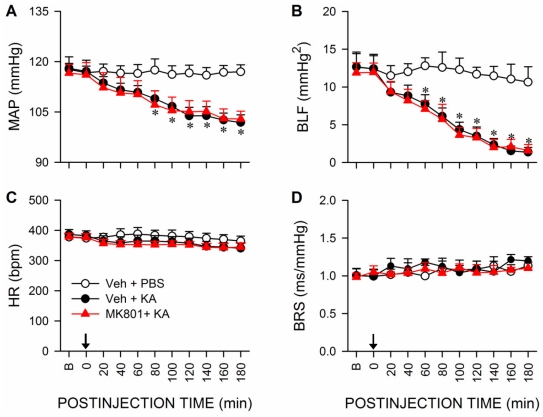
Glutamatergic neurotransmission in RVLM is not involved in the reduced baroreflex-mediated sympathetic vasomotor tone during experimental TLSE. Temporal changes in MAP (A), power density of the BLF component (B), HR (C) or BRS (D) after microinjection of KA (0.5 nmol) or PBS into the left hippocampal CA3 subfield (at arrow) of animals that received pretreatment by application into the bilateral RVLM of distilled water (Veh) or MK-801 (500 pmol). Values are mean ± SEM from 5–7 animals per experimental group. No significant difference exists (*P*>0.05) between treatment groups in two-way ANOVA with repeated measures.

Pretreatment by microinjection of a NMDA antagonist [44], MK-801 (500 pmol) into bilateral RVLM, similar to the vehicle, did not result in significant alterations of the reduction in MAP and power density of BLF component of SBP spectrum during experimental TLSE (Figure 12). MK-801 pretreatment also exerted minimal effects on the insignificant changes in HR or BRS (Figure 12).

## Discussion

Based on complementary physiological, pharmacological and biochemical evaluations, in associated with a clinically-compatible animal model, the present study revealed that upregulation of AT1R, which leads to oxidative stress that results in the formation of peroxynitrite by a reaction between the augmented superoxide anion and NOS II-derived NO in RVLM, underpins the reduction in baroreflex-mediated sympathetic vasomotor tone that takes place during experimental TLSE. Furthermore, upregulation of BDNF and TrkB in RVLM ameliorates this brain stem cardiovascular dysregulation via an antioxidant action.

A majority of studies that evaluates baroreflex functions measures primarily the associated cardiac responses; much less attention is paid to the vasomotor responses. It is therefore of interest that, by concurrently evaluating both forms of responses, the present study demonstrated that a significant reduction in baroreflex-mediated sympathetic vasomotor tone, which precedes significant hypotension, takes place during experimental TLSE. Intriguingly, the capacity for baroreflex-mediated sympathetic and vagal regulation of cardiac functions remains intact. An important functional implication of those observations is that baroreflex-mediated responses in heart and blood vessels do not necessarily have to take place in a concomitant fashion as generally assumed. More than 90% of SE-induced mortality does not occur during the seizures, but after a time lag following the seizure episode [45], [46]. The mechanism of this extended period of risk is not completely understood. The present study revealed that it is likely that HR is sustained during experimental TLSE because of the maintained baroreflex modulation of the heart. Deterioration of this homeostatic mechanism in the face of the already dysregulated baroreflex-mediated sympathetic vasomotor tone over time may offer a mechanistic underpinning for the delayed mortality following SE.

The present study revealed that oxidative stress in RVLM underlies the reduction in baroreflex-mediated sympathetic vasomotor tone during experimental TLSE. Specifically, our results indicated that activation of p47^phox^ subunit of NADPH oxidase is responsible for the augmented superoxide anion level in RVLM. NADPH oxidase is a major source for Ang II-elicited superoxide anion production in RVLM [47], [48], and its activation is a multistep process that is initiated by serine phosphorylation of the cytosolic regulatory p47^phox^ subunit [48], [49]. Our observed upregulation of AT1R in RVLM, along with an antagonism of the elevated superoxide anion, phosphorylated p47^phox^ subunit and progressive reduction in MAP or power density of BLF component of SBP spectrum by losartan, tempol or apocynin therefore suggests that the repertoire of cellular events in RVLM during experimental TLSE includes activation of AT1R, followed by augmented level of superoxide anion generated by the triggered p47^phox^ subunit of NADPH oxidase that leads to a reduction in baroreflex-mediated sympathetic vasomotor tone.

Oxidative stress in RVLM is known to be associated with the hypertensive state in animal models [50]. This notion seemingly contradicts our observed progressive reduction in MAP and baroreflex-mediated sympathetic vasomotor tone during experimental TLSE that is causally related to an elevation in superoxide level in RVLM. A potential solution to this paradox arises from two pieces of information from our previous work. First, NOS II is tonically active in RVLM [51]. Second, formation of peroxynitrite by a reaction between NOS II-derived NO and superoxide anion in RVLM results in a progressive decrease in MAP and the power density of the BLF component [52]. This solution is substantiated by the significantly elevated NOS II and nitrotyrosine (an experimental index of peroxynitrite) in RVLM observed during experimental TLSE. It follows that it is the formation of peroxynitrite because of the upregulated superoxide anion and NOS II-derived NO in RVLM that underpins the reduction in baroreflex-mediated sympathetic vasomotor tone, which leads to the decrease in MAP during experimental TLSE. On the other hand, a hypertensive state that is associated with oxidative stress in RVLM will prevail because of the significantly less NOS II expression and activity in RVLM of spontaneously hypertensive rats [53]. It should be noted that the occurrence of oxidative stress during experimental TLSE is not restricted to RVLM. Employing the same experimental model, we showed previously [32] that prolonged seizure prompted an increase in superoxide anion in the hippocampal CA3 subfield. Interestingly, on reacting with the simultaneously elevated NOS II-generated NO, the resultant formation of peroxynitrite elicits apoptotic cell death in hippocampal CA3 neurons by reducing the activity of mitochondrial respiratory enzyme Complex I.

Our observed transcriptional upregulation of *bdnf* and *trkB* genes suggests that de novo synthesis of BDNF and TrkB in RVLM may account for their augmented protein levels in RVLM during experimental TLSE. BDNF can enter the central nervous system by a rapid, saturable transport system of the blood-brain-barrier [54]. Thus, the elevated serum BDNF concentration may further contribute to its augmented level in RVLM. The present study also revealed that the upregulated BDNF and TrkB exert an antioxidant action in RVLM that leads to amelioration of brain stem cardiovascular dysregulation during experimental TLSE. The differential results from treatments with TrkB-Fc and TrkA-Fc further suggest that the antioxidant effect of BDNF against oxidative stress in RVLM is mediated via TrkB. Our laboratory demonstrated recently [29] that under chronic conditions, Ang II induces superoxide-dependent upregulation of BDNF in RVLM via phosphorylation of cAMP response element binding protein. The Ang II-activated BDNF/TrkB signaling, in turn, suppresses tissue superoxide level in RVLM through inhibition of p47^phox^ phosphorylation. The present study revealed that it is unlikely that this negative-feedback mechanism takes place in RVLM during acute seizure. Pretreatment with losartan, tempol or apocynin did not affect significantly the augmentation of BDNF/TrkB signaling in RVLM during the first 3 h after the induction of experimental TLSE.

Our results did support the notion that the upregulated BDNF/TrkB signaling exerts its antioxidant action via inhibition of p47^phox^ phosphorylation. We further demonstrated that by decreasing the production of superoxide anion, BDNF and TrkB in effect reduce the formation of peroxynitrite in RVLM without affecting NOS II expression. Our laboratory reported recently that activation of extracellular signal-regulated kinase (ERK) by BDNF/TrkB signaling [55], followed by mitogen-activated protein kinase signaling-interacting kinase [56] pathway in RVLM is responsible for ameliorating brain stem cardiovascular regulatory dysfunction during experimental brain death. More importantly and relevant to the present study, ERK is able to desensitize NADPH oxidase 1 (NOX 1) activity by phosphorylating NOX activator 1 at its serine 282 residue, resulting in the suppression of superoxide anion production [57]. It follow that ERK in the RVLM may also play a role in the antioxidant actions of BDNF/TrkB signaling during experiment TLSE by desensitizing the NADPH oxidase/superoxide cascade.

At the NTS, BDNF signaling exerts a tonic inhibitory modulation on primary afferent glutamatergic excitatory transmission and neural activity [28]. Of note is that acute activation of AT1R in RVLM potentiates glutamatergic neurotransmission [40]. Since our results indicated that the augmented BDNF/TrkB does not interact with the upregulated AT1R mRNA in RVLM at the level of transcription, it is possible that BDNF/TrkB signaling may also exert an inhibitory modulation on glutamatergic neurotransmission in RVLM during experimental TLSE. This possibility is deemed unlikely because we observed that glutamatergic neurotransmission in RVLM is not involved in the reduced MAP and baroreflex-mediated sympathetic vasomotor tone during experimental TLSE.

In conclusion, the present study presented a novel mechanistic view on the modus operandi whereby brain stem cardiovascular dysregulation predisposes mortality following SE. We showed that a reduction in baroreflex-mediated sympathetic vasomotor tone already occurs during the seizure episodes. Whether deterioration of the maintained baroreflex-mediated modulation of the heart ensues over time therefore becomes a crucial determinant for post-SE mortality to take place. We further showed that the repertoire of cellular events in RVLM that leads to reduction in baroreflex-mediated sympathetic vasomotor tone during experimental TLSE includes activation of AT1R, followed by augmented level of superoxide anion generated by the triggered p47^phox^ subunit of NADPH oxidase and NOS II, leading to the formation of peroxynitrite. Finally, the upregulated BDNF/TrkB signaling in RVLM ameliorates brain stem cardiovascular dysregulation by exerting an antioxidant action via inhibition of p47^phox^ phosphorylation. This information offers a new vista in devising therapeutic strategy or clinical management towards minimizing mortality associated with TLSE.

## Materials and Methods

### Ethics statement

All experimental procedures carried out in this study were approved by the Institutional Animal Care and Use Committee of the Kaohsiung Chang Gung Memorial Hospital (97005), and were in compliance with the guidelines for animal care and use set forth by that committee. All efforts were made to reduce the number of animals used and to minimize animal suffering during the experiment.

### Animals

Specific pathogen-free adult, male Sprague-Dawley rats (260 to 325 g, n = 253) purchased from the Experimental Animal Center of the National Science Council and BioLASCO, Taiwan, Republic of China were used. They were housed in an Association for Assessment and Accreditation of Laboratory Animal Care (AAALAC) International-accredited animal facility under temperature control (24–25°C) and 12-h light-dark cycle. Standard laboratory rat chow and tap water were available ad libitum.

### General preparation

Preparatory surgery, including tracheal intubation and cannulation of the femoral artery and vein was performed under an induction dose of pentobarbital sodium (50 mg/kg, i.p.). Rats received thereafter intravenous infusion of propofol (20–25 mg/kg/h; Zeneca, Macclesfield, England), which provided satisfactory maintenance of anesthesia while preserving the capacity of brain stem cardiovascular regulation [58]. During the recording sessions, animals were allowed to breathe spontaneously with room air, and body temperature was maintained at 37°C by a heating pad.

### Experimental temporal lobe status epilepticus

An experimental model [30]–[32] that mimics TLSE clinically was used. As in our previous study, KA (Tocris, Ellisville, MO, USA) dissolved in 0.1 M phosphate buffered saline (PBS, pH 7.4), at a concentration of 0.5 nmol, was microinjected stereotaxically (3.0–3.5 mm posterior to bregma, 1.4–2.2 mm from the midline, and 3.5–4.0 mm below the cortical surface) into the CA3 subfield of hippocampus on the left side. A small range of coordinates was adopted to adjust for the slight difference in brain size because of body weight of the animals. The volume of microinjection was restricted to 50 nl, and was delivered via a glass micropipette connected to a 0.5-µl microsyringe (Hamilton, Reno, NV, USA). Possible volume effect of microinjection was controlled by injecting the same amount of PBS in a separate group of animals. The hEEG signals were recorded from the right CA3 subfield by a stainless-steel bipolar concentric electrode (Rhodes Medical Instruments, Woodland Hills, CA; tip diameter: 100 µm), using the same stereotaxic coordinates as for microinjection. Bioelectrical signals were amplified and filtered (0.1 to 300 Hz) by a differential amplifier (A-M System, Sequim, WA, USA). The hEEG signals were simultaneously subject to continuous on-line and real-time spectral analysis (EEG10a, Notocord, Croissy-Sur-Seine, France). We quantified the magnitude of hEEG activity by calculating the RMS value. The frequency domain of hEEG signals was evaluated by calculating the MPF values. As a routine, hEEG signals and their RMS or MPF values were followed for 180 min after the induction of experimental TLSE.

### Recording of cardiovascular parameters and evaluation of baroreflex responses

AP recorded from the femoral artery was analyzed by an arterial blood pressure analyzer (APR31a, Notocord) to obtain SBP and PI. Continuous, on-line and real-time auto-spectral analysis (SPA10a, Notocord) of SBP signals was used to detect temporal fluctuations in the BLF (0.25–0.8 Hz) bands, the power density of which was used as the index for baroreflex-mediated sympathetic vasomotor tone [34]. To evaluate baroreflex-mediated cardiac responses, we employed a baroreflex sequence analyzer (BRS10a,) to determine the BRS based on on-line detection of spontaneous baroreflex sequences that were detected when SBP and PI increased or decreased simultaneously [35]. In addition, cross-spectral analysis of SBP and PI spectrum (CSA10a, Notocord) was used to reveal the gain of transfer function at the LF (0.25–0.8 Hz) and high-frequency (HF; 0.8–2.4 Hz) bands, which denotes respectively the efficacy of baroreflex-mediated sympathetic and parasympathetic regulation of cardiac functions [35]. Concurrent changes in the cardiovascular parameters and the four indices for baroreflex responses were followed for 180 min after the induction of experimental TLSE.

### Microinjection of test agents into RVLM

To produce site-specific pretreatments, test agents were microinjected bilaterally and sequentially into RVLM via a glass micropipette connected to a 0.5-µl Hamilton microsyringe [29], [37], [40], [48]. The coordinates used were: 4.5 to 5 mm posterior to the lambda, 1.8 to 2.1 mm lateral to the midline and 8.1 to 8.4 mm below the dorsal surface of the cerebellum. Again, a small range of coordinates was used to adjust for the slight difference in brain size because of body weight of the animals. As a routine, a total volume of 50 nl was delivered to each side of RVLM over 2–3 min to allow for complete diffusion of the test agents. Test agents used included a fluorescence indicator for superoxide anion [37], hydroethidine (Molecular Probes, Eugene, OR, USA); a superoxide dismutase mimetic [38], tempol (Calbiochem, San Diego, CA, USA); a specific antagonist of NADPH oxidase [39], apocynin (Calbiochem); an AT1R antagonist [41], losartan (Tocris); an AT2R antagonist [42], PD123319 (Tocris); a recombinant human TrkB-Fc fusion protein (R&D Systems, Minneapolis, MN, USA); a recombinant human TrkA-Fc fusion protein (R&D Systems); an antisense oligonucleotide against *bdnf* gene (5′-TCTTCCCCTTTTGGT-3′) and its sense control (5′-ACCAAAAGGGGAAGA-3′) (Quality Systems, Taipei, Taiwan); or a NMDA antagonist, MK-801 [44] (Sigma-Aldrich, St. Louis, MO, USA). The doses were adopted from our previous reports [29], [31], [32], [36], [40], [59] that used those test agents for the same purpose as in this study. Test agents were dissolved in 1% dimethyl sulfoxide (DMSO; Sigma-Aldrich) (hydroethidine, apocynin); distilled water (losartan, PD123319, tempol, antisense or sense oligonucleotide against *bdnf* gene, MK-801); or 0.1 M PBS (TrkB-Fc or TrkA-Fc fusion protein). Possible volume effect of microinjection was controlled by injecting the same amount of solvent. It should be mentioned that none of these solvents exhibited significant influence on MAP, HR, power density of BLF component of SBP spectrum and BRS (Table 1). All test agents or their vehicles were given 30 min before KA administration, with the exception that antisense or sense oligonucleotide was given 24 h prior to the induction of experimental TLSE. To avoid the confounding effects of drug interactions, each animal received only one test agent.

**Table 1:** Lack of effects of the solvents on baseline cardiovascular parameters and baroreflex responses.

Treatment		MAP (mmHg)	HR (bpm)	BLF (mmHg^2^)	BRS (ms/mmHg)
0.1 M PBS	Before	118.95±7.62	381.44±7.62	12.87±1.49	0.98±0.07
	After	117.75±1.91	376.47±13.8	11.63±1.36	1.01±0.08
Distilled water	Before	117.94±1.43	381.42±14.77	11.72±1.44	0.97±0.04
	After	118.12±2.00	385.85±15.95	12.37±1.99	1.04±0.07
1% DMSO	Before	118.75±2.01	383.9±13.68	11.81±1.37	0.99±0.05
	After	116.07±2.11	370.05±10.36	12.53±1.84	1.03±0.05

Values are mean ± SEM from 5–7 animals per experimental group, and were determined 30 min before and 30 min after experimental manipulations. No significant difference exists (*P*>0.05) between treatment groups in ANOVA.

### Measurement of superoxide anion

Superoxide anion in RVLM was determined semi-quantitatively by the intensity of fluorescence emitted by 2-hydroxyethidium, the specific reaction product between hydroethidine and superoxide. In brief, hydroethidine (1 mg/ml) was microinjected into the bilateral RVLM, 30 min before the commencement of experimental manipulations. At the conclusion of the experiment, animals were perfused transcardially with warm isotonic saline solution, followed by ice-cold 4% paraformaldehyde in 0.1 M PBS. The brain stem was removed, postfixed by submersion in the latter solution and cryoprotected by 30% sucrose in 0.1 M PBS. Frozen transverse sections of the medulla oblongata were cut on a cryostat (Leitz, Welzlar, Germany) and mounted on glass slides with K-Y jelly (Johnson & Johnson, New Brunswick, NJ, USA). Viewed under a laser scanning confocal microscope (FV10i, Olympus, Tokyo, Japan), the intensity of fluorescence relative to background in RVLM, which was designated the area of interest, was determined by an analysis program (Fluoview FV10-ASW Version.02.01; Olympus).

### Collection of tissue samples from RVLM

With minor exceptions, we routinely collected tissue samples from RVLM [29], [37], [40], [48] at 3 h after the induction of experimental TLSE. Medullary tissues collected from anesthetized animals but without treatment served as sham-controls. As a routine, microinjection sites were visually verified and recorded after the slice of medulla oblongata that contains RVLM (0.5 to 1.5 mm rostral to the obex) was obtained. Tissues from both sides of the ventrolateral medulla, at the level of RVLM were collected by micropunches made with a 1 mm (id) stainless steel bore to cover the anatomical boundaries of RVLM. The concentration of proteins extracted was determined by the BCA Protein Assay (Pierce, Rockford, IL, USA).

### Western blot analysis

Western blot analysis [29], [40], [48] was carried out using a rabbit polyclonal antiserum against TrkB (Novus Biologicals, Littleton, CO, USA), AT1R or AT2R (Chemicon, Temecula, CA, USA) or NOS II (Santa Cruz Biotechnology, Santa Cruz, CA, USA); or a mouse monoclonal antiserum against β-actin (Chemicon). This was followed by incubation with horseradish peroxidase-conjugated donkey anti-rabbit IgG (GE Healthcare, Little Chalfont, Buckinghamshire, UK) for TrkB, AT1R or AT2R; or sheep anti-mouse IgG (GE Healthcare) for β-actin. Specific antibody-antigen complex was detected by an enhanced chemiluminescence Western blot detection system (Santa Cruz Biotechnology). The amount of protein was quantified by the ImageMaster software (Amersham Pharmacia Biotech, Buckinghamshire, UK), and was expressed as the ratio relative to β-actin protein.

### Immunoprecipitation and immunoblot analysis

Protein extracts from the cytosolic fraction of samples from RVLM were immunoprecipitated with affinity-purified goat polyclonal anti-p47^phox^ antiserum conjugated with protein G-agarose beads. Immunoprecipitation was performed [29], [48] at 4°C overnight and the precipitated beads were washed three times with ice-cold lysis buffer. The agarose beads resuspended in the loading buffer were boiled for 10 min to dissociate the immunocomplexes from the beads. Western blot analysis of phosphoserine or p47^phox^ from proteins immunoprecipitated by anti-p47^phox^ antiserum was carried out as described above.

### Isolation of RNA and real-time PCR

Total RNA from RVLM was isolated with a Total RNA Mini kit (Geneaid, Taipei, Taiwan) according to the manufacturer's instructions. All RNA isolated was quantified by spectrophotometry and the optical density (OD) 260/280 nm ratio was determined. Reverse transcriptase reaction was performed using a Transcriptor First strand cDNA Synthesis kit (Roche, Mannheim, Germany). Real-time PCR analysis [29], [48] was performed by amplification of cDNA using a LightCycler (Roche). PCR reaction for each sample was carried out in triplicate for all the cDNA and for the GAPDH control. Primers were designed using the sequence information of the NCBI database by Roche LightCycler probe design software 2.0, and oligonucleotides were synthesized by Quality Systems (Taipei, Taiwan).

The primer pairs used for amplification of target genes were:

BDNF (Genbank Accession: NM_012513 ):

Forward primer: 5′-GTTAGGAGAAGTCAAGCTGGA-3′

Reverse primer: 5′-AAGCAATTGTTTGCCTCTTT-3′

TrkB (Genbank Accession: NM_001163168):

Forward primer: 5′-CATCTATCTACCTATCATGTCTGG-3′

Reverse primer: 5′-AATGTTGCTGAAATGGTTGTTAT-3′

AT1R (Genbank Accession: NM_030985):

Forward primer: 5′-CCTCTGACTAAATGGCTTACG-3′

Reverse primer: 5′-CATCTATTAATGCAAGACGGC-3′

AT2R (Genbank Accession: NM_012494):

Forward primer: 5′-TGGGAGTCTCTGACAGTTC-3′

Reverse primer: 5′-AAATGCTTATCTGCCGGT-3′

GAPDH (Genbank Accession: NM_017008):

Forward primer: 5′-CTTCTCTTGTGACAAAGTGGA-3′

Reverse primer: 5′-TTAGCGGGATCTCGCTC-3′

Fluorescence signals from the amplified products were quantitatively assessed using the LightCycler software program (version 3.5). The second derivative maximum mode was chosen with baseline adjustment set in the arithmetic mode. The relative changes in mRNA expression were determined by the fold-change analysis, in which Fold change = 2^−[ΔΔCt]^, where ΔΔCt = (Ct_gene_−Ct_GAPDH_)_KA treatment_−(Ct_gene_−Ct_GAPDH_)_sham control_). Note that Ct value is the cycle number at which fluorescence signal crosses the threshold.

### Determination of BDNF or nitrotyrosine in serum or RVLM

The level of BDNF in serum or tissues collected from RVLM was determined by a commercial ELISA kit (Millipore, Billerica, MA, USA). Nitrotyrosine in RVLM was similarly determined by a commercial ELISA kit (Cell Biolabs, San Diego, CA, USA). Quantified of BDNF or nitrotyrosine was carried out by measuring the absorbance at 450 nm in conjunction with spectrophotometry (Thermo Scientific, Waltham, MA, USA). Blood (200 µl) drawn from the femoral artery was centrifuged to obtain serum samples, which were frozen at −80°C until analysis.

### Statistical analysis

All values are expressed as mean ± SEM. The averaged value of MAP, HR, BRS, LF gain or HF gain calculated every 20 min after administration of test agents or vehicle, the sum total of power density for the BLF component in the SBP spectrum over 20 min, and changes in fluorescence intensity of 2-hydroxyethidium, real-time PCR products or protein expression in RVLM during experimental TLSE, was used for statistical analysis. One-way or two-way ANOVA with repeated measures was used, as appropriate, to assess group means. This was followed by the Scheffé multiple-range test for post hoc assessment of individual means. *P*<0.05 was considered to be statistically significant.
